# Sustained activity of novel THIOMAB antibody-antibiotic conjugate against *Staphylococcus aureus* in a mouse model: Longitudinal pharmacodynamic assessment by bioluminescence imaging

**DOI:** 10.1371/journal.pone.0224096

**Published:** 2019-10-29

**Authors:** Chenguang Zhou, Hao Cai, Amos Baruch, Nicholas Lewin-Koh, Meng Yang, Fengxun Guo, Deming Xu, Rong Deng, Wouter Hazenbos, Amrita V. Kamath

**Affiliations:** 1 Research and Early Development, Genentech Inc., South San Francisco, California, United States of America; 2 Product Development, Genentech Inc., South San Francisco, California, United States of America; 3 WuXi AppTec (Shanghai) Co., Ltd., Shanghai, China; Massachusetts General Hospital, UNITED STATES

## Abstract

*Staphylococcus aureus* (*S*. *aureus*) infections are a leading cause of death by an infectious agent. Survival within host phagocytic cells is one mechanism by which *S*. *aureus* evades antibiotic treatment. A novel THIOMAB^™^ antibody-antibiotic conjugate (TAC) strategy was developed to kill *S*. *aureus* intracellularly and mitigate the spread of infection. In this report, we used a longitudinal whole-body bioluminescence imaging method to study the antibacterial dynamics of TAC alone or in combination with vancomycin in a mouse infection model. Injections of stably luminescent *S*. *aureus* bacteria into mice resulted in exponential increases in whole body bioluminescence with a reduction in body weight and survival rate. Vancomycin, a standard-of-care antibiotic, suppressed bacterial growth in mice. However, bacterial growth rebounded in these animals once treatment was discontinued. In contrast, single dose of TAC showed rapid reduction of bioluminescence intensity, which persisted for up to 19 days. The combination of TAC and vancomycin achieved a more sustained and significantly greater reduction of bioluminescence compared with vancomycin alone. In summary, the present study showed an imaging method to longitudinally assess antibacterial drug dynamics in mice and demonstrated that TAC monotherapy or in combination with vancomycin had superior and sustained activity compared to vancomycin alone.

## Introduction

*Staphylococcus aureus* (*S*. *aureus*) is a leading cause of human bacterial infections [1, 2], with infected patients exhibiting severe complications including infective endocarditis, pyelonephritis, and osteomyelitis [3, 4]. Antibiotics, such as vancomycin and nafcillin, have been used as standard of care for treating *S*. *aureus* infections [5]. However, these antibiotics are associated with a relatively high failure rate in the treatment of invasive *S*. *aureus* infection. One possible mechanism is that *S*. *aureus* can be internalized and survive within phagocytes, thereby establishing an intracellular reservoir that can further spread infection. While standard of care antibiotics are efficient against planktonic bacteria, much higher extracellular concentrations of antibiotics are required to kill intracellular *S*. *aureus* bacteria than to inhibit growth of extracellular bacteria [3, 4, 6]. Therefore, a therapeutic agent that targets persistent intracellular bacteria for invasive *S*. *aureus* disease may show improved clinical outcomes [7].

A THIOMAB antibody-antibiotic conjugate (TAC) was developed to kill intracellular *S*. *aureus* [8, 9] and is currently being evaluated in Phase 1b clinical trials. The TAC molecule consists of a monoclonal human immunoglobulin (Ig)G1 antibody that specifically binds to wall teichoic acids (a cell wall antigen) of *S*. *aureus*, a protease cleavable valine-citrulline linker, and a novel antibiotic 4-dimethylamino piperidino-hydroxybenzoxazino rifamycin (dmDNA31), which is a potent rifampin-class antibiotic with an in vitro minimum inhibitory concentration (MIC) of < 10 nM against methicillin-resistant S. aureus USA300 in neutral (pH = 7.4) [8] and acidic environment (pH = 5.0). When binding to *S*. *aureus* in circulation, the antibody portion of TAC facilitates the uptake of bacteria into phagocytes through opsonization. In the phagolysosome, the TAC linker is cleaved by proteases such as cathepsins, thereby releasing the active antibiotic that kills the bacteria bound to TAC as well as pre-existing intracellular bacteria in the same phagocytes. In addition, since TAC exhibits a longer systemic half-life compared to small molecule antibiotics [8], the sustained concentrations of TAC in the circulation can capture *S*. *aureus* released from intracellular reservoirs, mitigating the spread of infection.

In our previous study, we evaluated the pharmacodynamics (PD) of TAC using *S*. *aureus*-infected severe combined immunodeficiency (SCID) mice [9]. The study showed that single intravenous (IV) administration of TAC significantly reduced the bacterial load (reflected by a decrease of bacterial colony forming units, CFU) in multiple organs [9]. This approach that is based on terminal CFU counting is not amenable to real-time observation of *S*. *aureus* infection progression longitudinally in each individual animal. This CFU counting method would require large numbers of animals for tissue collection at multiple time points, which is not only labor-intensive but can also cause high intra and inter study variability.

Bioluminescence imaging has been reported as an alternative approach to evaluate the progression of gram positive and negative bacterial infections. Mice systemically or locally injected with bioluminescent bacteria have been used to noninvasively monitor progression of infection caused by *Escherichia coli* [10], *Pseudomonas aeruginosa* [11], etc. To achieve live imaging of *S*. *aureus* infection and assess antibacterial activities of antibiotics, several types of bioluminescent *S*. *aureus* were developed [12, 13, 14, 15, 16]. For example, Plaut et al. [14] generated stably luminescent *S*. *aureus* via genetic transformation of a clinical strain by a series of genes encoding enzymes for luminescent reaction in naturally light-emitting organisms. Mice injected with these genetically engineered bacteria were used as model for live imaging of *S*. *aureus* infection. Total body luminescence intensity in infected mice was continuously recorded via imaging and used to represent bacterial load. These previous studies also suggested that stably luminescent bacteria maintained the characteristics of their parental clinical strains, had similar virulence [14] and were responsive to treatment by standard of care antibiotics [13].

In the present study in infected mice, we applied a bioluminescence imaging approach with stably luminescent bacteria to assess 1) longitudinal dynamics of bacterial load and disease progression in each individual animal, 2) PD of TAC as monotherapy or in combination with standard of care, and 3) dose-response relationship that drives the antibacterial activities of TAC.

## Materials and methods

### Material

Stably luminescent *S*. *aureus* clinical strain (SAP149) was generously provided by Dr. Roger Plaut from US Food and Drug Administration [14]. The anti-*S*. *aureus* TAC was generated at Genentech Inc. (South San Francisco, CA). The TAC molecule is composed of a human IgG1 monoclonal antibody that targets wall teichoic acids, a protease cleavable linker (maleimido-caproyl-valine-citrulline-para-aminobenzyl), and the payload antibiotic, piperazino. Piperazino was conjugated to the antibody at the cysteine residues with an average antibiotic to unconjugated antibody ratio of ~2 accordingly [8]. The conjugation method was described in previous publications [8, 17, 18]. In brief, the Val 205 position of the anti-wall teichoic acid antibody light chain was engineered and conjugated to linker-piperazino. SAP149 bacteria (~5.0×10^8^ CFU/mL) were suspended in 50% glycerol and stored at -80°C. To prepare the inoculum, bacterial stocks were thawed on ice and diluted in PBS to the desired amount.

### Mouse infection model

Female CB-17 SCID mice were purchased from Vital River Laboratory Animal Company (Beijing, China). All animal studies were performed in the WuXi AppTec (Shanghai) Co., Ltd. animal facility accredited by the Association for Assessment and Accreditation of Laboratory Animal Care International. All the protocols and procedures for animal studies were approved by WuXi AppTec Institutional Animal Care and Use Committee (IACUC) and performed in accordance with institutional and regulatory guidelines (Protocol Number: R20141229-mouse). All animals were monitored on a daily basis for body weight changes and additional clinical signs of bacterial infection e.g., changes in body posture and coat appearance, reduction in activity and food/water intake. Due to the nature of mouse bacterial infection model, the only adverse effects observed were infection-related symptoms. Since the purpose of the study is to evaluate the efficacy of TAC against bacterial infection progression, no other antibiotic or analgesia was used to manage the infection to avoid interference with TAC. Instead, if any infected animals lose >20% of the original body weight or reach moribund status, they were removed from the study immediately, and euthanized by CO2 inhalation. No special housing conditions were given. All the personnel involved in animal handling, injections, and care were trained according to IACUC guideline.

To generate the mouse infection model, stably luminescent bacteria were inoculated to 6–8 weeks old female SCID mice via the lateral tail vein injection. Different numbers of bacteria (from 2.2×10^6^ to 3.5×10^7^ CFU per mouse) were first injected to animals to establish a correlation between bioluminescence intensity and CFU and identify an appropriate amount of inoculated bacteria for efficacy study. For efficacy study, an inoculation amount of 1×10^7^ CFU bacteria was injected to animals.

In total, 210 mice were used (including backup mice) for all experiments described. Among these animals, 30 animals were euthanized as humane endpoints were reached, and 20 animals were found dead before euthanasia. The cause of death was bacterial infection. All the remaining animals were euthanized at the end of the experiment. The maximum duration of each experiment is 31 days.

### In vivo efficacy study

Mice were treated with saline, vancomycin (94747, Sigma Aldrich) alone, TAC alone, or vancomycin plus TAC at one day post bacterial inoculation (12 animals per treatment group) to be consistent with clinical scenarios. Vancomycin was administered to mice b.i.d. for 7 days intraperitoneally. TAC was injected IV to mice via lateral tail vein as a single dose. It was administered at a dose of 15, 50, or 100 mg/kg as monotherapy and 5, 15, or 50 mg/kg when combined with vancomycin. The dose of TAC and vancomycin was determined based on the previous published results [8, 9, 19, 20].

### Bioluminescence imaging

Mice were anesthetized with an intraperitoneal injection of 75 mg/kg of sodium pentobarbital (P276000, Toronto Research Chemicals) and transferred to an imaging chamber. Mice were imaged from dorsal or ventral view using Xenogen IVIS Lumina II system equipped with the charge-coupled device (CCD) camera (PerkinElmer, MA). Bioluminescence images were quantified using the Xenogen Corperation Living Image software (PerkinElmer, MA). The total intensity was calculated from a user-defined area of interest covering the whole animal body except the paws. Lower limit of quantification of imaging is approximately 1×10^8^ photons (p).

### CFU measurement

Organs including kidney, liver, spleen, and heart were isolated after animals were euthanized. The whole organ tissues were excised, and transferred into 10 ml saline, and homogenized using a digital disperser (IKA Works Inc., NC). The homogenates were serially diluted and plated on tryptic soy agar plates at 37°C for 24 h. The bacterial burden in the organs was determined by counting colonies in the highest dilution and multiplying the colony number with the dilution factor.

### Statistical analyses

All experiments were performed on biological replicates. Sample size for each experimental group was reported in the figure legends. Linear as well as nonlinear regression (i.e. exponential plateau equation) was applied to determine the relationship between CFUs from each organ and the total bioluminescence intensity. Log-rank (Mantel-Cox) test was performed to determine the statistical significance in the comparison of survival rates. Nonparametric Kruskal-Wallis test followed by multiple comparisons was performed to determine statistical significance in the comparison of CFUs in organs from mice treated with saline *versus* vancomycin. One-way analysis of variance (ANOVA) followed by Dunnett’s test was used to determine the statistical significance in the comparison of area under the bioluminescence intensity-time curve (AUC _bioluminescence intensity_) from mice on vancomycin treatment *versus* saline or TAC treatments. A cut-off p-value < 0.05 was used as the criteria of statistical significance for all the analyses. The statistical analyses were performed using Prism (GraphPad Inc., CA).

## Results

### Bioluminescence signal intensity correlated with bacterial load and disease progression

**Fig 1:**
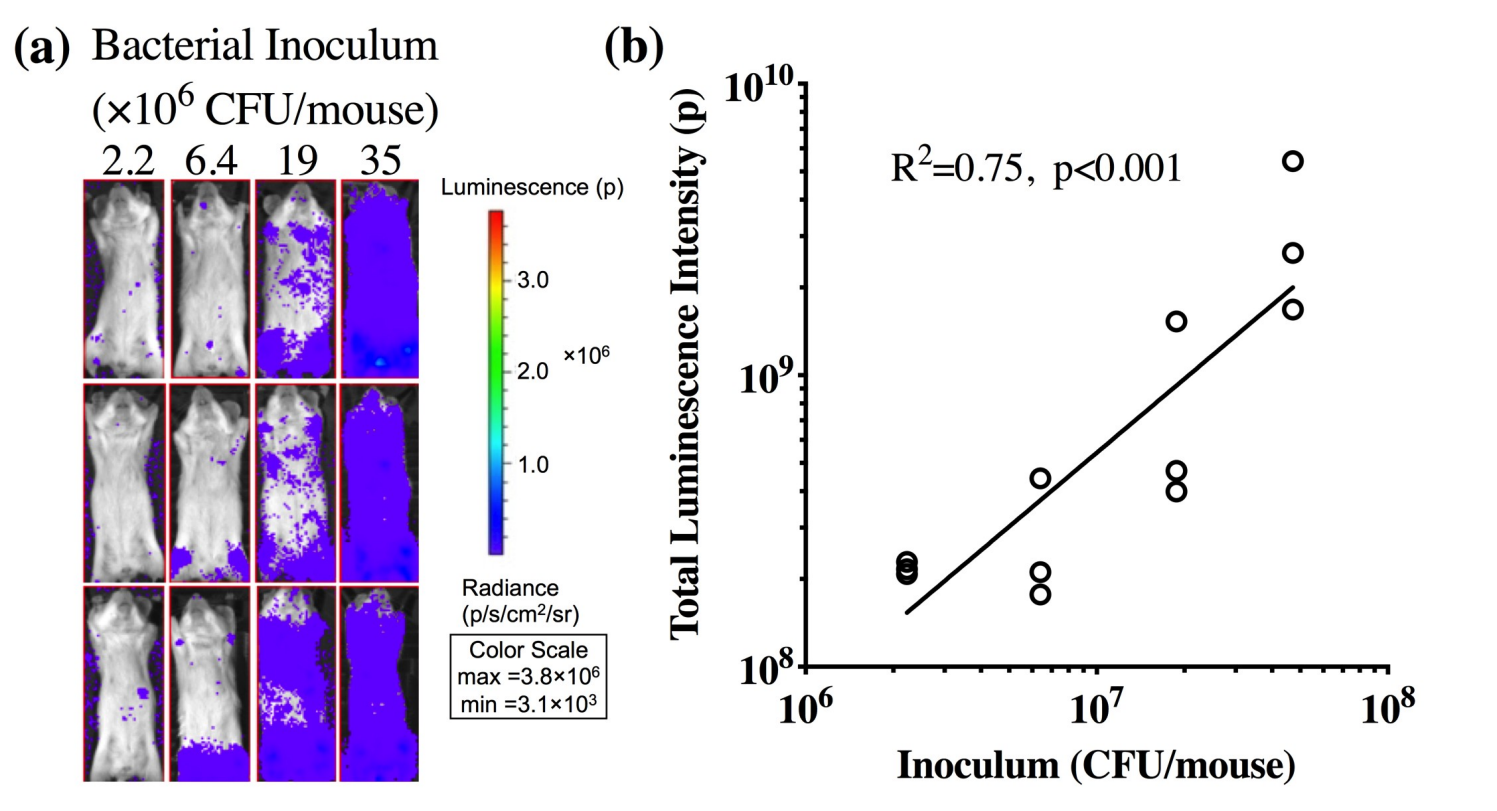
Correlation between bacterial inoculum and total luminescence intensity at one day post bacteria inoculation. (**a**) Representative bioluminescence images of mice inoculated with different bacterial amounts (N = 3 per group) as indicated. (**b**) Correlation analysis between quantified luminescence intensity and bacterial inoculum.

**Fig 2:**
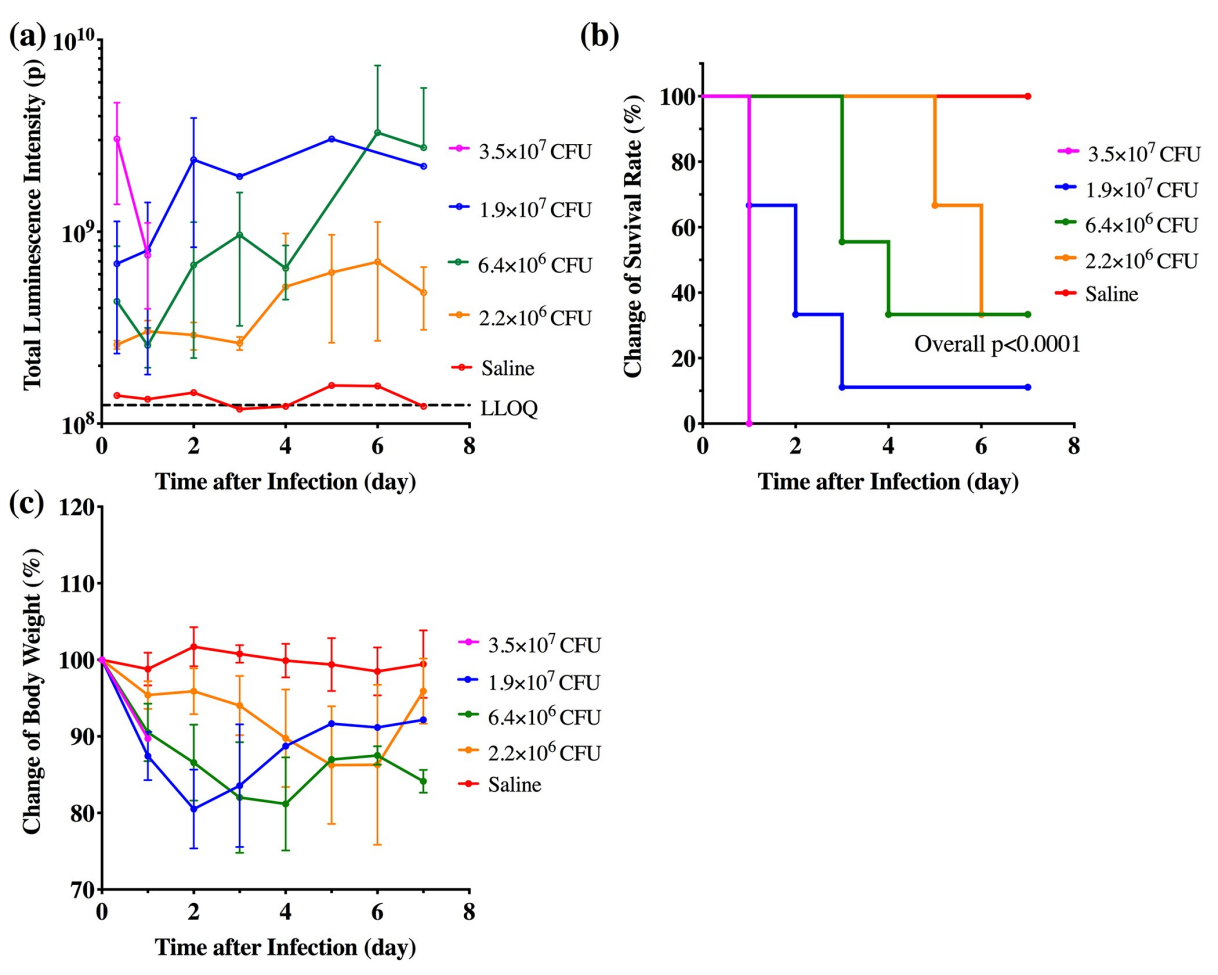
Dynamics of infection progression in mice inoculated with saline (N = 3 per group) or different bacterial amounts (N = 9 per group). (**a**) Bioluminescence intensity-time profile in mice inoculated with different bacterial amounts. (**b**) Kaplan-Meier curves of survival rate and (**c**) body weight in mice with different bacterial inoculums. Data are represented as mean ± SD. Error bar was not added to the body weight curve of 1.9x10^7^ CFU/mouse group after Day 3 as there was only one surviving animal left.

To investigate if bioluminescence signal can be used to quantitatively measure bacterial load and to optimize the number of bacteria used to generate the infected mouse model, different amounts of stably luminescent bacteria (i.e. 2.2×10^6^, 6.4×10^6^, 1.9×10^7^ or 3.5×10^7^ CFU/mouse) were inoculated into the mice. Dorsal imaging at one day post inoculation demonstrated a strong linear correlation between the bioluminescence intensity and inoculated bacteria amount (p<0.001, R^2^ = 0.75, Fig 1). Continuous increase in bioluminescence intensity in the infected mice was observed through the one-week follow-up period post inoculation (Fig 2A). No change of bioluminescence intensity was observed in non-infected animals. Bioluminescence intensity observed from dorsal view was comparable to the observed from ventral view suggesting efficient signal penetration (S1 Fig).

The previous study by Plaut, et al., [14] showed that stably luminescent bacterial infection in mice resulted in a reduction of survival rate, which is a symptom of mice infected with clinical strains of *S*. *aureus*. We took a next step by assessing if the total body luminescence signal can be an indicator of disease progression. Increase in total body bioluminescence intensity seen with higher bacterial amounts was associated with increased mortality and decreased body weight (Fig 2B and 2C). While all animals inoculated with PBS (control) survived during the 7-day observation, even lowest amount of bacterial inoculation (i.e. 2.2×10^6^ CFU/mouse) caused 70% reduction of animal survival rate in the end of study. All animals inoculated with highest bacterial amount (3.5×10^7^ CFU/mouse) died at one-day post inoculation (Fig 2B). Consistent with survival study, a 10–20% reduction of body weight was observed in mice inoculated with bacteria compared to the control group (Fig 2C).

These data suggested that bioluminescence imaging method, was able to quantitatively monitor bacterial growth and infectious disease progression. In addition, the results enabled us to identify appropriate inoculation amount for 3-week efficacy study next. A medium bacterial inoculum of 1×10^7^ CFU/mouse (S2 Fig) was used, because a higher inoculum (e.g. 1.9×10^7^ CFU/mouse) would cause fast disease progression hampering long-term observation, while a lower bacterial inoculum (e.g. 2.2×10^6^ CFU/mouse) would result in a lower bioluminescence signal precluding accurate quantification.

### Bioluminescence model captured the treatment effect of standard of care antibiotic

**Fig 3:**
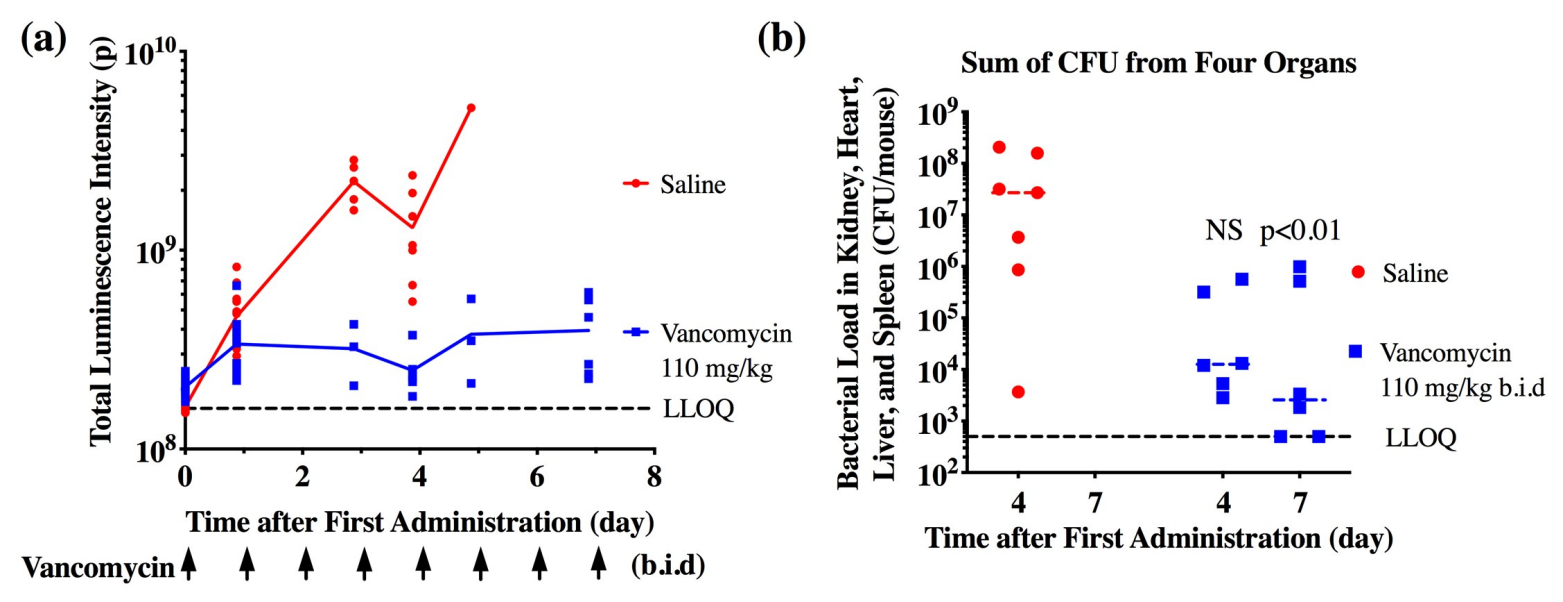
Quantitative evaluation of vancomycin pharmacodynamics (N = 12 per group). (**a**) Bioluminescence intensity-time profile in mice treated with saline or vancomycin (110 mg/kg, b.i.d, for 7 days). (**b**) Bacterial load measured in the combination of four organs (i.e. heart, kidney, spleen, and spleen) isolated from mice subjected to saline or vancomycin treatment. Seven animals on saline treatment and six animals on vancomycin treatment were euthanized on Day 4. All animals from saline groups were euthanized before Day 7 due to disease progression and therefore, no data points are shown. All the remaining animals from vancomycin treatment were euthanized on Day 7. NS: not significant.

The feasibility of using the bioluminescent bacterial infection model for longitudinal real-time evaluation of antibacterial activity of a therapeutic agent was assessed using the standard of care antibiotic vancomycin. An amount of 1×10^7^ CFU luminescent bacteria was injected into each mouse to achieve an optimized disease progression rate as discussed above. These mice were treated with saline (control group) or 110 mg/kg vancomycin twice a day (b.i.d.) and imaged for 7 days. Both vancomycin and TAC treatment started at one day post inoculation to match the clinical scenarios. This dose and treatment regimen of vancomycin reflects dosing frequency and exposure in humans [19]. While an exponential growth of bacteria in saline-treated animals was observed, bioluminescence intensity remained unchanged in mice during the course of treatment with vancomycin, suggesting a potent antimicrobial treatment effect (Fig 3A).

**Fig 4:**
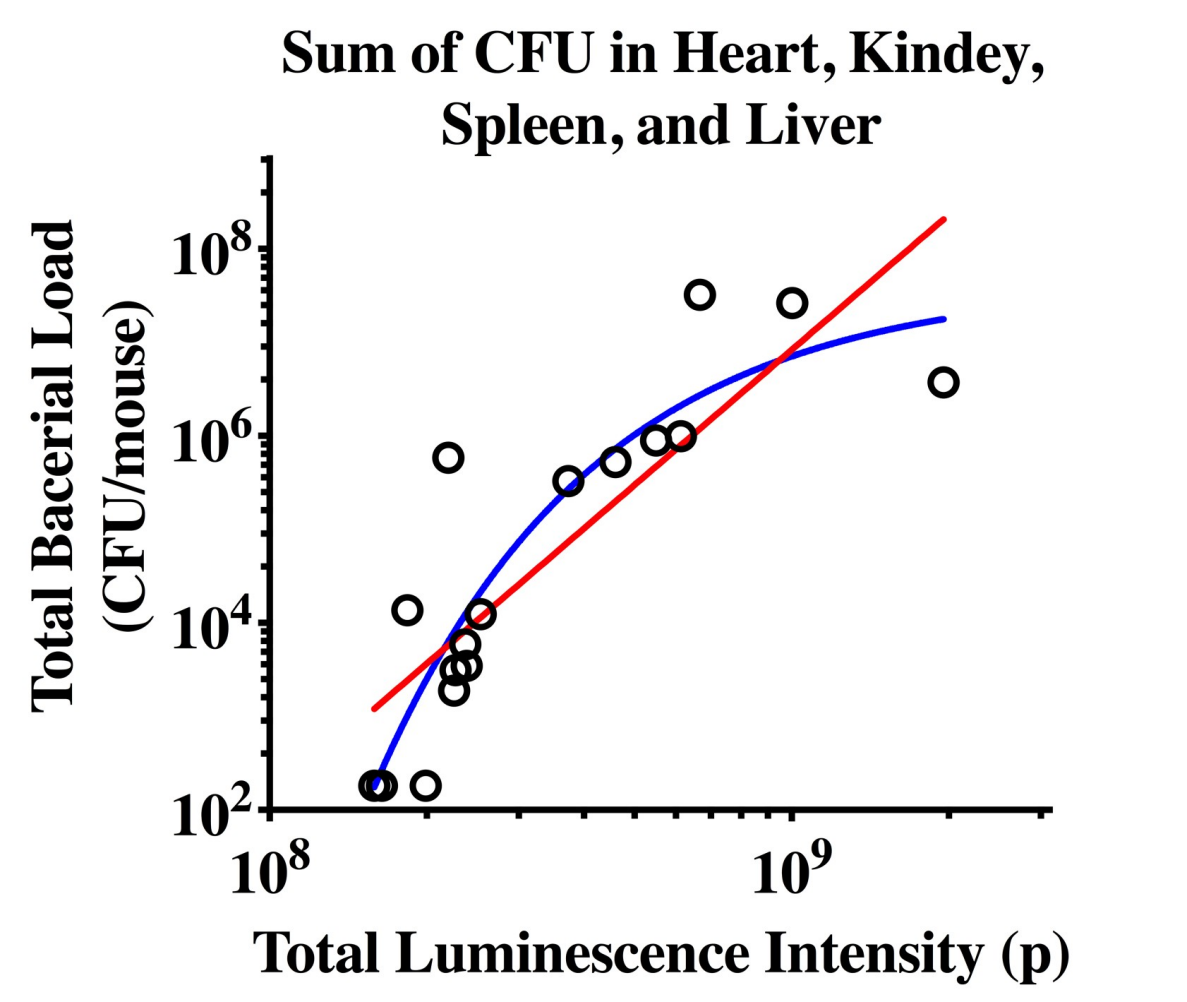
Correlation of bioluminescence intensity with CFU from the combination of four organs (i.e. heart, kidney, spleen, and liver). Animals on saline or vancomycin treatment (110 mg/kg, b.i.d, for 7 days) were euthanized after the last administration. Bioluminescence intensity and CFU were measured. Both linear (red) and nonlinear regression (blue) were applied. N = 16 in total.

The correlation between total bioluminescence intensity and CFU was then assessed on Day 4 or Day 7 and CFUs from multiple organs were determined (Fig 3B). A significant reduction of CFU was observed in the sum of four organs at the end of vancomycin treatment compared to the saline control group. Correlation was observed between total bioluminescence intensity and CFU from the kidney (p<0.05, R^2^ = 0.61) and liver (p<0.05, R^2^ = 0.38), but not heart (p = 0.64, R^2^ = 0.03) or spleen (p = 0.65, R^2^ = 0.03) (S3 Fig). Total bioluminescence intensity was best correlated with sum of CFUs from all these organs (p<0.0001, R^2^ = 0.71) (Fig 4). This could be explained as bioluminescence intensity was obtained via whole body imaging rather than from each individual organ. As the bacterial load increase with bioluminescence intensity might be saturable, the relationship between bioluminescence intensity and bacterial load was also fit using a nonlinear regression method.

### Single dose of TAC showed long-term inhibition of bacterial growth

**Fig 5:**
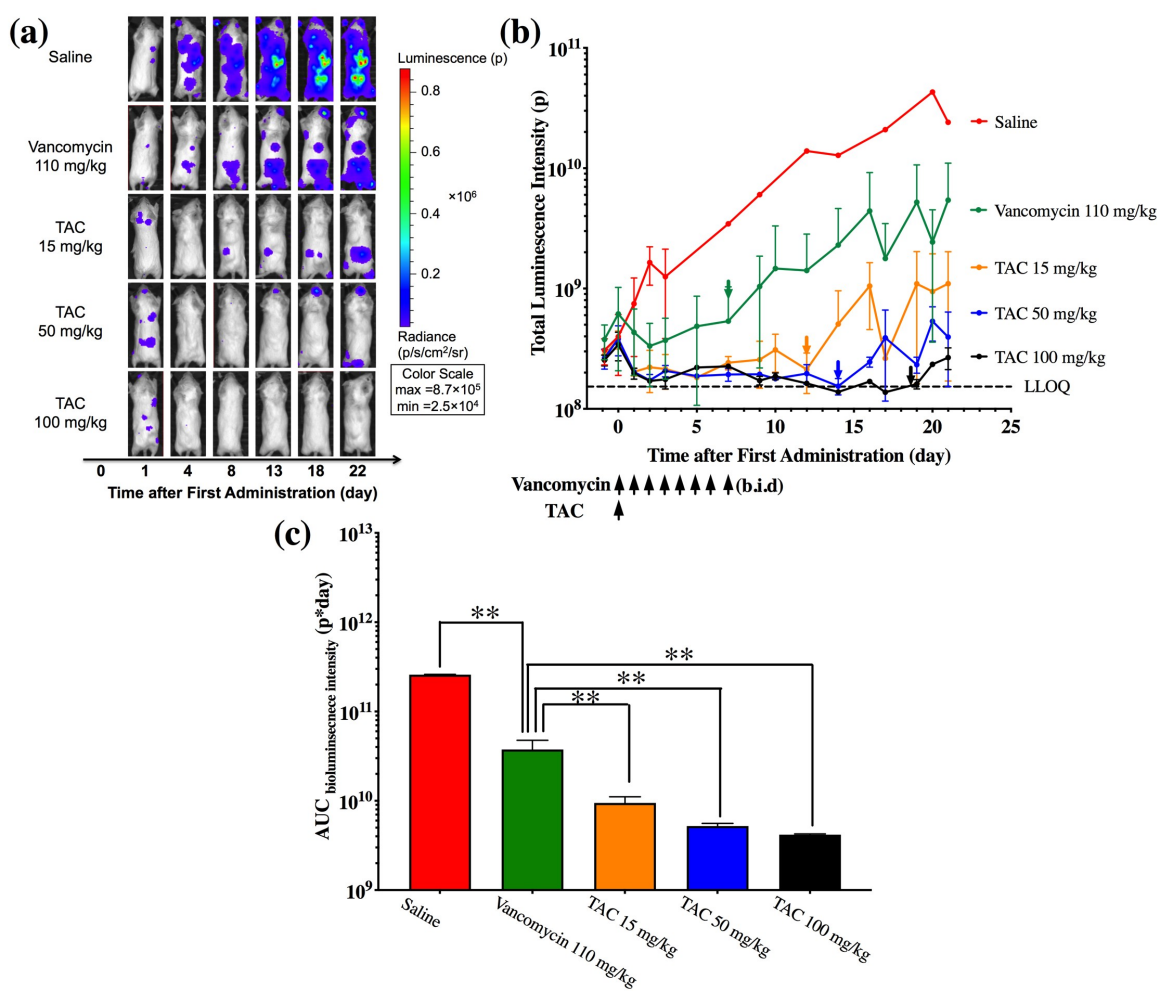
Assessment of antibacterial efficacy of vancomycin or TAC as monotherapy. (**a**) Representative bioluminescence images of infected mice subjected to saline, vancomycin (110 mg/kg, b.i.d., for 7 days), or TAC (15, 50, 100 mg/kg, once) treatments. (**b**) Bioluminescence intensity-time profile of infected mice on different treatments. The time when bacterial growth begins to rebound is indicated by the colored arrows. The days when vancomycin or TAC is administered are also pointed. (**c**) AUC _bioluminescence intensity_ from mice on different types of treatment. Data are represented as mean ± SD (N = 12 per group). **p<0.01.

Bioluminescence imaging was next applied to evaluate the PD profile of TAC. One day post inoculation with 1.0×10^7^ CFU stably luminescent bacteria, mice were treated with 110 mg/kg vancomycin b.i.d. for 7 days or with a single IV dose of TAC at three dose levels of 15, 50, or 100 mg/kg. An exponential increase in total bioluminescence intensity was observed in mice on saline treatment (Fig 5A and 5B). Treatment with vancomycin stabilized the bacterial load in mice during the treatment period. However, the bacterial load rebounded once the treatment ended, with a comparable growth rate (reflected by the slope of bioluminescence intensity-time curve) as the saline-treated group (Fig 5B). Strikingly, a single dose of TAC, at all three dose levels tested, not only caused a rapid reduction in bioluminescence intensity (Fig 5B) but also achieved sustained inhibition of bacterial growth for at least 12 days post infection compared to vancomycin monotherapy, reflected by a significant reduction (p<0.01 for TAC at all three dose levels) of AUC _bioluminescence intensity_ (Fig 5B and 5C). The inhibitory effect of TAC on bacterial growth was dose-dependent, as the time to bacterial re-growth was prolonged from 12 to 19 days when the dose was increased from 15 to 100 mg/kg (Fig 5B). In addition, we observed significantly lower mean total luminescence intensity in the end of study in animals with TAC at 100 mg/kg dose compared to 15 mg/kg (2.67×10^8^ p *vs*. 1.10×10^9^ p, p<0.05). A trend toward dose-dependent reduction of AUC _bioluminescence intensity_ was also observed (Fig 5C). Animals on TAC monotherapy at different dose levels also showed improved survival rate compared to animals on vancomycin (S4 Fig). The extended antibacterial activity of TAC can be attributed to maintaining sustained drug concentrations after a single administration due to its long half-life [9].

### TAC plus vancomycin enhanced the antibacterial efficacy of vancomycin

**Fig 6:**
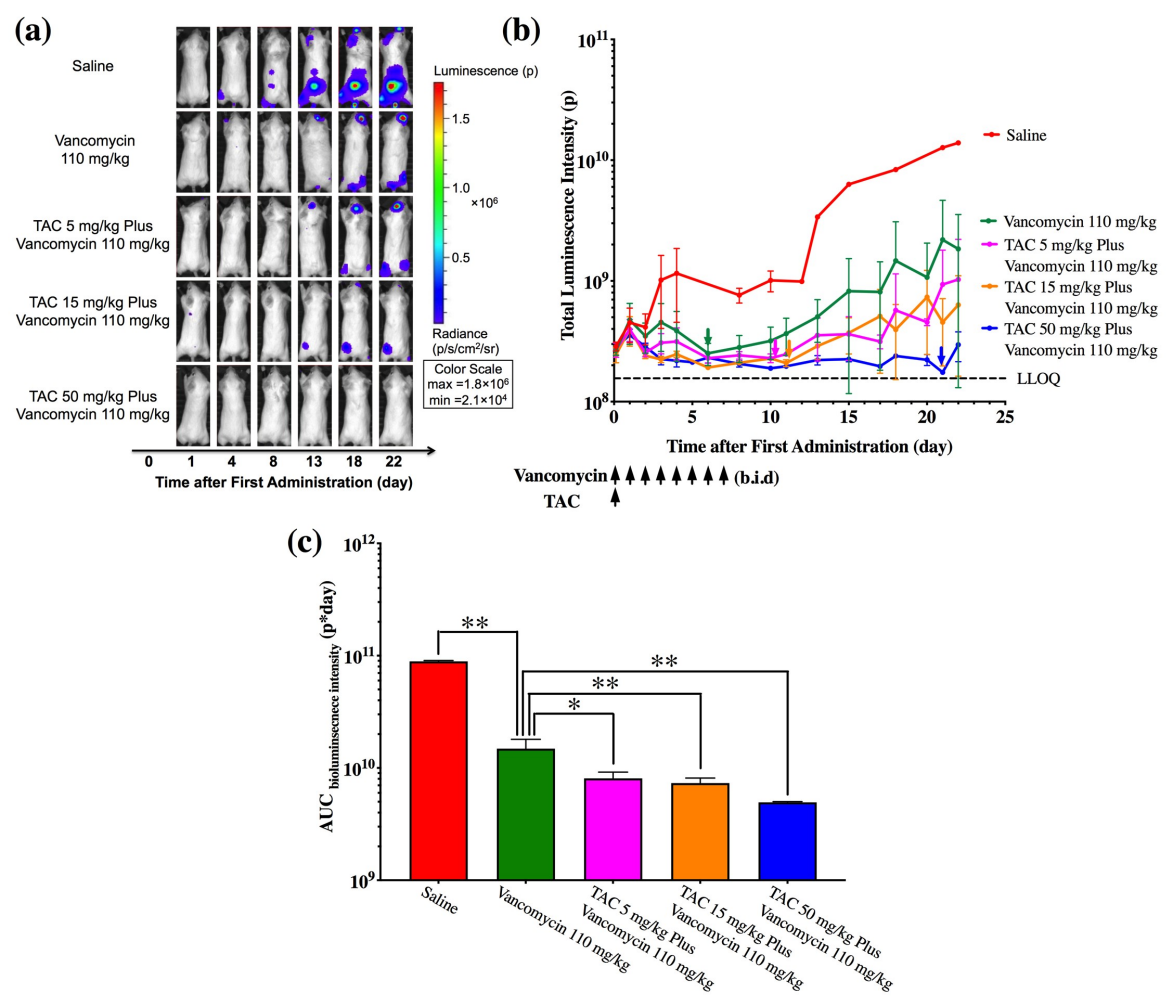
Assessment of antibacterial efficacy of vancomycin in combination with TAC. (**a**) Representative bioluminescence images of infected mice subjected to saline, vancomycin (110 mg/kg, b.i.d, 7 days), or vancomycin (110 mg/kg, b.i.d, 7 days) plus TAC (5, 15, 50 mg/kg, once) treatments. (**b**) Bioluminescence intensity-time profile of infected mice on different treatments. The time when bacterial growth begins to rebound is indicated by the colored arrows. The days when vancomycin or TAC is administered are pointed. (**c**) AUC _bioluminescence intensity_ from mice on different types of treatment. Data are represented as mean ± SD (N = 12 in each group). *p<0.05. **p<0.01.

Since antibacterial efficacy of TAC at different dose levels plus standard of care antibiotic will be compared to standard of care antibiotic alone in current ongoing phase 1 study (NCT03162250), we then investigated the PD of combination therapy of TAC and vancomycin in the bioluminescence imaging model to understand the potential of combining TAC with standard of care antibiotics in patients. As described in the method section, infected mice were treated with saline, 110 mg/kg vancomycin b.i.d. for 7 days, or 110 mg/kg vancomycin b.i.d. for 7 days plus a single dose of TAC at either 5, 15, or 50 mg/kg (Fig 6). Consistent with the monotherapy, combination treatment of TAC and vancomycin caused a significant reduction of AUC _bioluminescence intensity_ compared to vancomycin alone (p<0.05 for 5 mg/kg dose level, p<0.01 for 15 and 50 mg/kg dose levels) (Fig 6C). Bioluminescence intensity data showed that TAC plus vancomycin exhibited enhanced antibacterial activity compared to monotherapy of vancomycin or TAC at the same dose level. Combination treatment of 50 mg/kg TAC and vancomycin achieved a longer bacterial growth suppression compared to 50 mg/kg TAC monotherapy (21 *vs*. 14 day) (Figs 5B and 6B). Compared to TAC monotherapy at the same dose level, combination treatment of 50 mg/kg TAC and vancomycin showed a trend of lower total luminescence intensity (2.96 ×10^8^ *vs*. 3.95×10^8^ p), although the difference was not statistically significant (S5 Fig). These observations demonstrate that TAC in combination with vancomycin results in greater antibacterial efficacy compared to vancomycin alone and could provide a superior therapeutic outcome to control *S*. *aureus* infections.

## Discussion

Treatment of invasive *S*. *aureus* infections with standard of care antibiotics is associated with relatively high failure rates. This may be due to the presence of specific reservoirs of viable *S*. *aureus* bacteria that are protected against the action of these antibiotics and cause relapses. Indeed, it has been well established that *S*. *aureus* bacteria are relatively protected against a number of clinically relevant antibiotics when residing intracellularly in macrophages [6]. To overcome this challenge, the TAC molecule was designed to specifically release payload antibiotic inside phagocytes and kill *S*. *aureus* intracellularly [8]. In our previous study using SCID mice infected with *S*. *aureus*, we have demonstrated that single administration of TAC significantly reduced bacterial load in heart, liver, and kidney [9]. However, we were not able to monitor the impact of TAC on disease progression in each individual animal. This is because tissues would need to be isolated from euthanized animals, and a longitudinal assessment would require a large number of animals and higher inter-individual variation.

To overcome the limitations of conventional CFU measurement in infectious mouse model, the bioluminescence approach was established to measure antibacterial efficacy against multi-resistant *S*. *aureus* by using stably luminescent bacteria-infected mice [12, 13, 14, 15, 16]. We performed a systematic investigation of whether bioluminescence imaging can provide longitudinal real-time PD assessment of TAC.

We first validated whether stably luminescent bacteria exhibited physiological and pathological behaviors similar to clinical strains. Consistent with published results [13], an exponential increase in bioluminescence intensity was observed in the infected animals in the saline treated control group. The increase in bioluminescence intensity correlated with body weight loss and reduction of survival rate of the mice. Similar to clinical strains of *S*. *aureus*, stably luminescent bacteria were also sensitive to standard of care antibiotic, e.g. vancomycin treatment, as vancomycin suppressed the increase in bioluminescence intensity in the infected animals.

A previous study by Mortin et al [13], suggested bioluminescence intensity might reflect bacterial load in tissues, as a correlation between bioluminescence intensity and CFU from mouse thigh was observed. The present study provides a further evaluation on whether bioluminescence intensity could be a quantitative indicator of bacterial load in multiple organs. Bioluminescence intensity only correlated with CFUs from kidney and liver but not heart and spleen. One explanation is that injected bacteria established foci of infection mainly in the kidney, liver and what appear as lymph nodes. As a result, total bioluminescence intensity was mainly contributed by the luminescence signal from kidney and liver, and subsequently could reflect the CFUs from both organs. Organs such as heart and spleen had relatively low bacterial counts and only marginally added to the overall bacterial load. Therefore, the total bioluminescence intensity was not sensitive enough to the minor difference in the CFU from heart and spleen among animals. As expected, best correlation was observed when bioluminescence intensity was compared with the sum of CFUs from different organs since the bioluminescence intensity was obtained via whole body imaging.

Some studies have suggested that the correlation between bioluminescence intensity and bacterial load might be changed in mice on different antibacterial treatments as antibiotic pressure can enhance bioluminescence signals [21, 22]. Therefore, caution needs to be applied when directly comparing the bioluminescence intensity between animals on different antibiotic therapies.

Although studies have shown stably luminescent bacteria are responsive to small molecule antibiotics, it is not clear whether mice infected with stably luminescent bacteria can be an appropriate model to compare antibacterial activity of a novel modality, antibody-antibiotic conjugate. The present study showed single administration of TAC at dose levels from 15 to 100 mg/kg caused a rapid reduction of bioluminescence intensity and the reduction was sustained for up to 19 days. In contrast, vancomycin was only able to suppress the bacteria load during treatment, and bacterial growth rebounded with a rate comparable with saline treated animals once the treatment was stopped. This observation can be explained by a more thorough bacterial killing in tissues and a longer systemic half-life of TAC *versus* vancomycin. Combination treatment of vancomycin and TAC exerted greater and more prolonged antibacterial activities compared with vancomycin alone. The results suggested that bacterial killing caused by standard of care antibiotics such as vancomycin, does not affect the efficacy of TAC and an additional benefit can be obtained if TAC is added to current standard of care. The data support testing TAC in combination with standard of care antibiotics in patients.

The present study also evaluated the feasibility of using the bioluminescence imaging approach to establish a dose-response relationship for TAC. The bioluminescence imaging model provided additional information that is critical for PD evaluation of TAC compared to conventional CFU counting. Although a previous study also showed reduction of bacteria in multiple organs by TAC, the conventional approach used in that study did not differentiate the responses to different TAC dose levels [9]. The bioluminescence imaging model showed that although treatment of TAC with different dose levels exhibited similar reduction of bioluminescence intensity in the first week, the duration of bacterial growth suppression was dose-dependent. By increasing the dose from 15 to 100 mg/kg, the duration of suppression of bacterial growth was prolonged from 12 days to 19 days and the AUC _bioluminescence intensity_ was reduced from 9.46×10^9^ to 4.16×10^9^ p*day. One explanation is the drug concentrations can be maintained over a longer duration at the 100 mg/kg dose compared to 15 mg/kg (as shown in the simulated PK profiles in S6 Fig).

In addition to the ability of surviving in phagocytes or other host cells, forming biofilm is believed to be another cause of multidrug resistance of *S*. *aureus*. *S*. *aureus* can form a multilayered bacterial community embedded within glycocalyx or protein matrix, which prevents the efficient diffusion of antibiotics [23]. Further studies are needed to evaluate the effect of TAC on *S*. *aureus* biofilm in appropriate models [24, 25].

## Conclusions

We show that the bioluminescence imaging approach can be used to provide a longitudinal assessment of antibacterial dynamics of a novel therapeutic modality (i.e. antibody-antibiotic conjugate) in each individual mouse, which is difficult to obtain by terminal CFU counting. Studies in mice infected with stably luminescent bacteria demonstrated that TAC exhibited a dose-dependent sustained suppression of bacteria. Compared to standard of care vancomycin monotherapy, TAC, either as a monotherapy or in combination with vancomycin, exerted superior and more sustained antibacterial activity.

## Supporting information

S1 Fig Comparison of bioluminescence intensity readout by imaging from ventral *versus* dorsal site. (TIFF)

S2 Fig Disease progression of mice injected with 1×10^7^ CFU/mouse bioluminescent bacteria. (**a**) Change of body weight in mice and (**b**) Kaplan-Meier curves of survival rate. Data are represented as mean ± SD. N = 12 per group.
(TIFF)

S3 Fig **Correlation of bioluminescence intensity with CFU from (a) heart, (b) kidney, (c) spleen, and (d) liver.** Animals on saline or vancomcin treatment (110 mg/kg, b.i.d, for 7 days) were euthanized after the last administration and tissues were isolated for CFU counting and luminescence intensity measurement.
(TIFF)

S4 Fig Survival rate of mice with TAC monotherapy or in combination with vancomycin. (**a**) Kaplan-Meier curves of survival rate in infected mice treatment with saline, vancomycin (110 mg/kg, b.i.d, 7 days), or TAC (15, 50, 100 mg/kg, once). (**b**) Kaplan-Meier curves of survival rate in infected mice treatment with saline, vancomycin (110 mg/kg, b.i.d, 7 days), or vancomycin (110 mg/kg, b.i.d, 7 days) plus TAC (15, 50, 100 mg/kg, once).
(TIFF)

S5 Fig Comparison of antibacterial efficacy between TAC monotherapy and TAC in combination with vancomycin. The bioluminescence data are from studies presented as Figs 5 and 6. The days when vancomycin or TAC is administered are pointed. Data are represented as mean ± SD (N = 12 in each group).
(TIFF)

S6 Fig Simulated plasma concentration of antibody-conjugated dmDNA31 in mice with single IV administration of TAC at 5, 15, 50 and 100 mg/kg. The simulated plasma concentrations were obtained using a two-compartment PK model, which was established by fitting the model to the reported data from previous single dose mouse PK study [8]. All PK parameter calculations and simulations were performed using WinNolin 6.4 (Pharsight, Mountain View, CA).
(TIFF)
