# Supplementary figures and images for: Sustained activity of novel THIOMAB antibody-antibiotic conjugate against Staphylococcus aureus in a mouse model: Longitudinal pharmacodynamic assessment by bioluminescence imaging
Source: PLoS One. 2019 Oct 29;14(10):e0224096. doi: 10.1371/journal.pone.0224096 (PMC6818770; doi:10.1371/journal.pone.0224096)

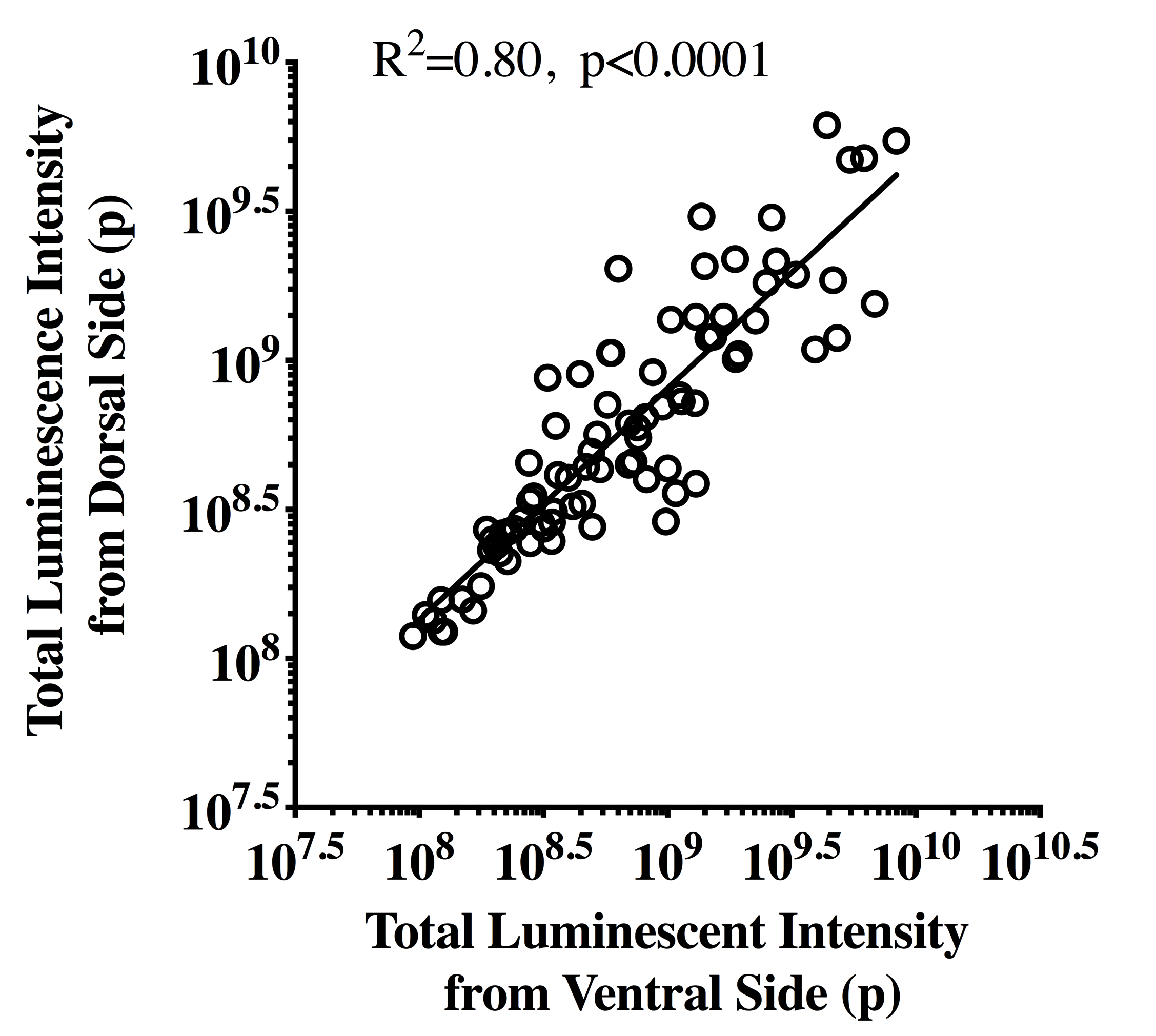

Supplement: S1 Fig — (TIFF) [file pone.0224096.s001.tiff]

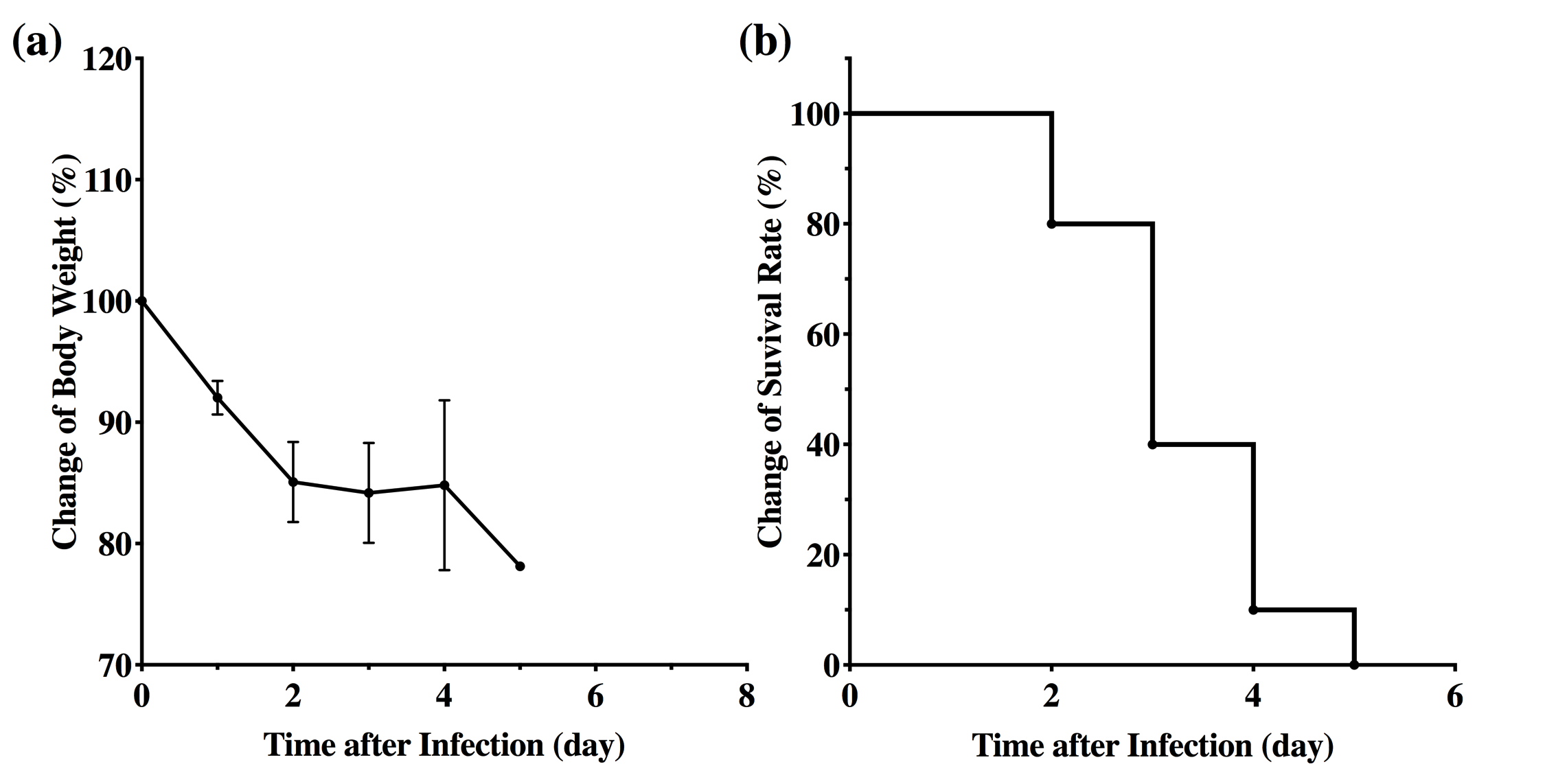

Supplement: S2 Fig — (a) Change of body weight in mice and (b) Kaplan-Meier curves of survival rate. Data are represented as mean ± SD. N = 12 per group. (TIFF) [file pone.0224096.s002.tiff]

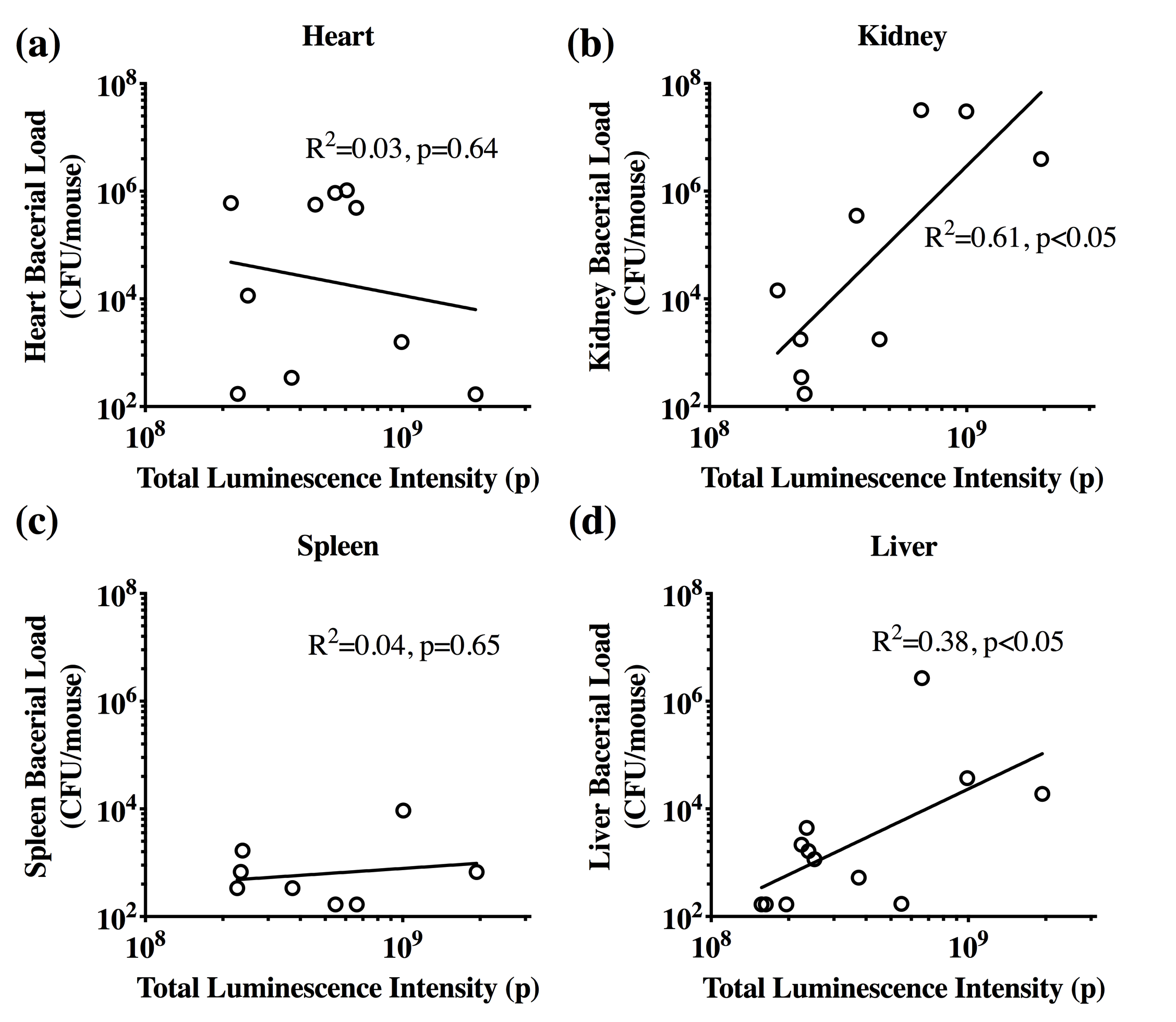

Supplement: S3 Fig — Correlation of bioluminescence intensity with CFU from (a) heart, (b) kidney, (c) spleen, and (d) liver. Animals on saline or vancomcin treatment (110 mg/kg, b.i.d, for 7 days) were euthanized after the last administration and tissues were isolated for CFU counting and luminescence intensity measurement. (TIFF) [file pone.0224096.s003.tiff]

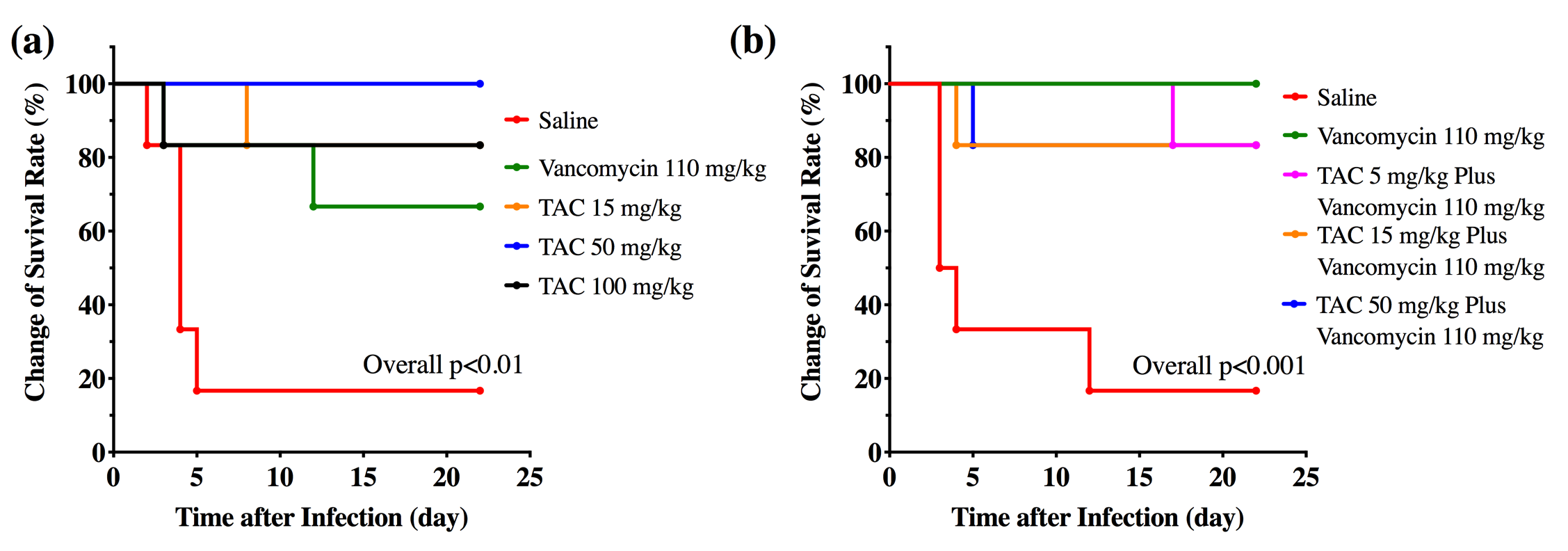

Supplement: S4 Fig — (a) Kaplan-Meier curves of survival rate in infected mice treatment with saline, vancomycin (110 mg/kg, b.i.d, 7 days), or TAC (15, 50, 100 mg/kg, once). (b) Kaplan-Meier curves of survival rate in infected mice treatment with saline, vancomycin (110 mg/kg, b.i.d, 7 days), or vancomycin (110 mg/kg, b.i.d, 7 days) plus TAC (15, 50, 100 mg/kg, once). (TIFF) [file pone.0224096.s004.tiff]

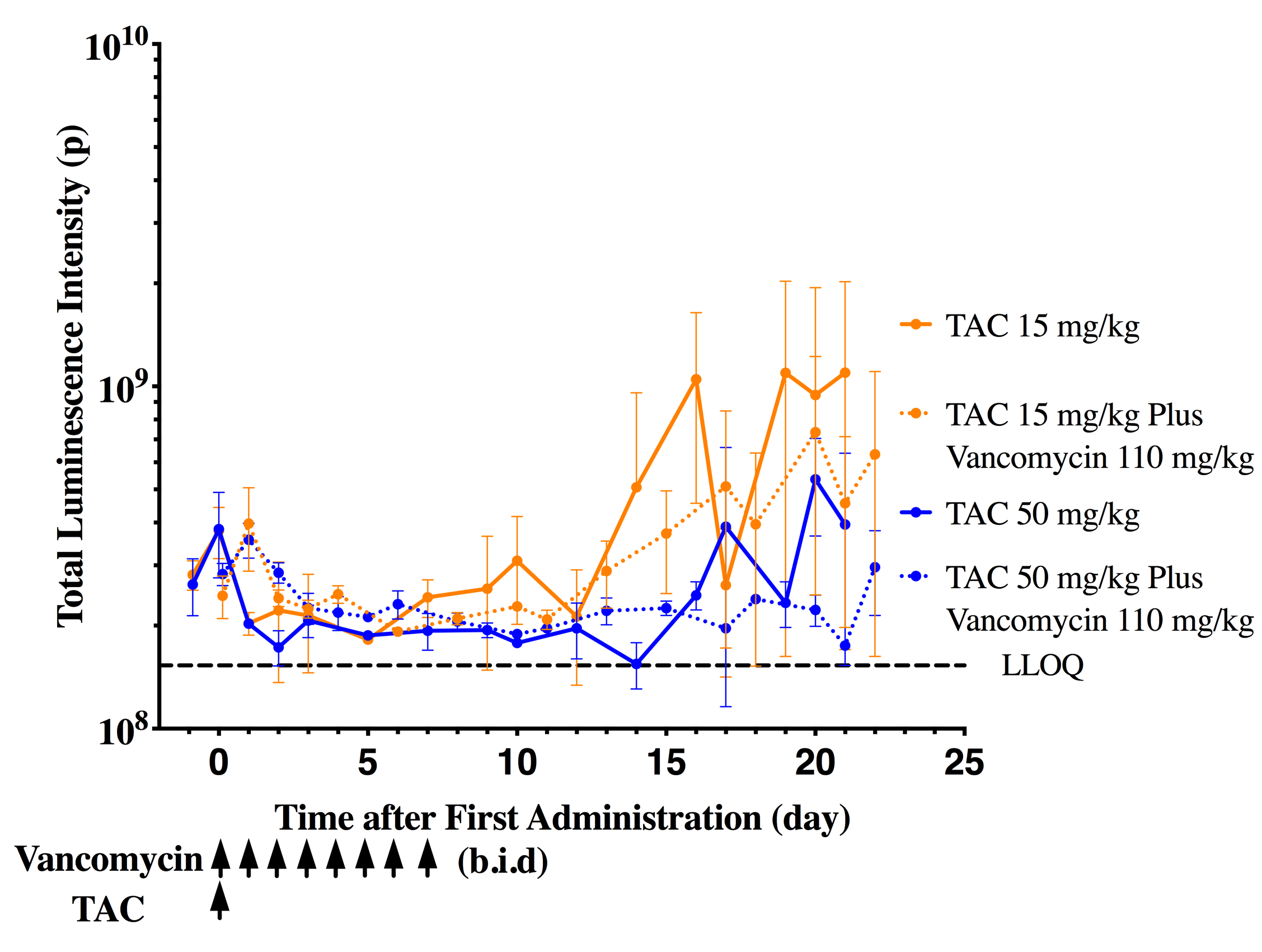

Supplement: S5 Fig — The bioluminescence data are from studies presented as Figs 5 and 6. The days when vancomycin or TAC is administered are pointed. Data are represented as mean ± SD (N = 12 in each group). (TIFF) [file pone.0224096.s005.tiff]

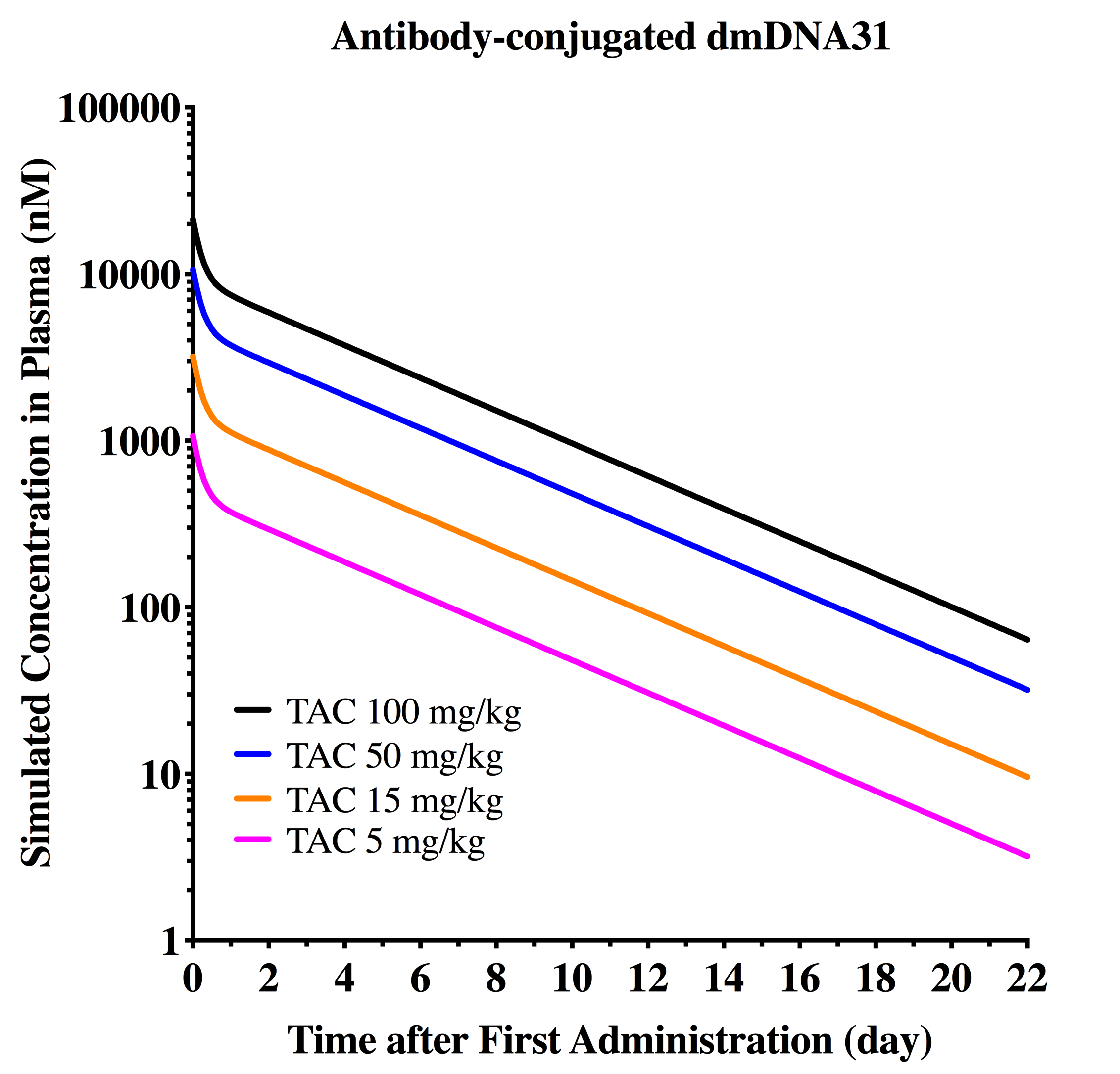

Supplement: S6 Fig — The simulated plasma concentrations were obtained using a two-compartment PK model, which was established by fitting the model to the reported data from previous single dose mouse PK study [8]. All PK parameter calculations and simulations were performed using WinNolin 6.4 (Pharsight, Mountain View, CA). (TIFF) [file pone.0224096.s006.tiff]
